# Feasibility of a Noncontact Photoplethysmography–Based Mobile App for Noninvasive Hemoglobin Monitoring: Exploratory Observational Study

**DOI:** 10.2196/78820

**Published:** 2026-02-19

**Authors:** Gianvincenzo Zuccotti, Paolo Agnelli, Lucia Labati, Erika Cordaro, Davide Braghieri, Paolo Fiorina, Simona Bertoli, Cesare Celeste Federico Berra, Marina Croci, Maria Elena Malighetti, Fabrizio Losurdo, Andrea Foppiani, Loredana Bucciarelli, Marco Xodo, Alberto Battezzati, Sergio De Pasquale, Valeria Calcaterra

**Affiliations:** 1 Department of Biomedical and Clinical Science, University of Milan, Via GB Battista 32, Milan, 20154, Italy, 39 0263635321; 2 Pediatric Department, Buzzi Children’s Hospital, Milan, Italy; 3 Come Stai S.p.A., Milan, Italy; 4 Department of Internal Medicine, Pediatric and Adolescent Unit, University of Pavia, Pavia, Italy; 5 Division of Endocrinology, ASST Fatebenefratelli-Sacco, Milan, Italy; 6 Department of Biomedical and Clinical Science, International Center for T1D, Pediatric Clinical Research Center Romeo ed Enrica Invernizzi, University of Milano, Milan, Italy; 7 Department of Food, Environmental and Nutritional Sciences (DeFENS), International Center for the Assessment of Nutritional Status and the Development of Dietary Intervention Strategies (ICANS-DIS), University of Milan, Milan, Italy; 8 Department of Endocrine and Metabolic Diseases, Laboratory of Nutrition and Obesity Research, IRCCS Istituto Auxologico Italiano, Milan, Italy; 9 Dipartimento Cardiovascolare e Metabolico, IRCCS MultiMedica, Sesto San Giovanni-Milano, Milan, Italy; 10 Ambulatorio di Diabetologia, Casa di Cura Ambrosiana, Cesano Boscone, Milan, Italy; 11 Department of Endocrine and Metabolic Medicine, Clinical Nutrition Unit, IRCCS Istituto Auxologico Italiano, Milan, Italy; 12 Pio Albergo Trivulzio, Milan, Italy

**Keywords:** noncontact photoplethysmography, noncontact PPG, mobile app, hemoglobin, noninvasive monitoring

## Abstract

**Background:**

Anemia is a widespread global health issue. Hemoglobin (Hb) concentration measurement remains the most common method for anemia screening and diagnosis. In recent years, there has been growing interest in the development of noninvasive point-of-care technologies that eliminate the need for blood sampling.

**Objective:**

This pilot study explores the feasibility of using a noncontact photoplethysmography–based mobile app for Hb monitoring.

**Methods:**

Adult volunteers aged 18 years and older, of both sexes, were consecutively recruited. Participants were seated and allowed a 2-minute rest before measurements. During testing, they faced a smartphone running comestai.app*,* which used the front-facing camera to capture facial videos. Simultaneous readings were collected for Hb over approximately 90 seconds using the app. Ambient lighting was standardized for all remote photoplethysmography recordings. No medical decisions were made based on the app-generated data. A complete blood count, including Hb levels, was used as a reference for comparison with the data collected using comestai.app.

**Results:**

A total of 555 (female: n=313, 56.4%; male: n=242, 43.6%) individuals participated in the study. The app achieved a mean absolute error of 1.46, a mean absolute percentage error of 11.26, a mean error of −0.67, and a root mean square error of 1.88. The Bland-Altman plot evaluated the agreement between the app-based and laboratory-based Hb measurements, with the mean difference between the 2 methods being −0.70 g/dL. The method demonstrated an overall accuracy of 75%. The area under the curve was 0.701 (95% CI 0.655-0.745).

**Conclusions:**

Comestai.app offers an innovative approach to wellness monitoring by providing noninvasive Hb estimation using the smartphone’s front-facing camera. Continued development, including algorithmic refinement and larger-scale validation in diverse populations, will be key to enhancing accuracy and broadening its utility. By leveraging the ubiquity of smartphones, comestai.app contributes meaningfully to the democratization of health monitoring and the promotion of proactive self-care.

## Introduction

### Background

Anemia is a widespread global health issue and a major public health concern, particularly in resource-limited settings [1]. It affects individuals across all age groups and sexes, leading to adverse health outcomes and reduced national economic productivity [2]. According to the World Health Organization (WHO), anemia is estimated to affect approximately half a billion women aged 15 to 49 years and 269 million children aged 6 to 59 months worldwide. In 2019, an estimated 30% (539 million) of nonpregnant women and 37% (32 million) of pregnant women within this age group were affected by anemia [1,3].

Anemia can result from various underlying causes, including nutritional deficiencies (such as iron, vitamin B12, or folate), chronic blood loss, chronic or infectious diseases, genetic disorders, bone marrow and hematologic conditions, as well as exposure to toxins and certain medications [1]. Early and accurate detection of anemia is crucial for effective patient management and improved health outcomes [4].

Hemoglobin (Hb) concentration measurement remains the most common method for anemia screening and diagnosis [2,4-6]. Conventional techniques typically involve invasive procedures such as venipuncture or fingerstick blood tests, requiring trained personnel, laboratory infrastructure, and costly equipment [7-9]. These methods, while accurate, pose logistical challenges in remote settings, contribute to patient discomfort, introduce delays in reporting, and generate biomedical waste [7-9].

To address these limitations, point-of-care (POC) devices have been developed to facilitate rapid Hb estimation using capillary blood from a simple finger prick [3,10,11]. While such devices improve field usability, they still require reagent stability, periodic calibration, and controlled storage conditions [10,11].

In recent years, there has been a growing interest in the development of noninvasive POC technologies that eliminate the need for blood sampling [12-14]. These innovations aim to address the limitations of traditional and minimally invasive methods by enhancing usability, portability, and affordability, particularly in low-resource settings. Devices such as digital hemoglobinometers, pulse oximeters, and spectroscopy-based technologies have shown considerable promise in expanding access to anemia screening [15-17].

Among these, photoplethysmography (PPG)-based devices represent a particularly attractive approach [18,19]. By measuring variations in light absorption due to pulsatile blood flow, PPG enables indirect estimation of Hb concentration without requiring blood collection or chemical reagents, thereby increasing safety and convenience for both patients and health care workers.

In this context, smartphone-based technologies are also emerging as promising tools for noninvasive anemia detection using PPG signals [20-23]. By using the built-in camera and flash, several apps estimate Hb concentration through image analysis of anatomical regions such as the fingernail beds, palpebral conjunctiva, or lower eyelids. These systems often use machine learning (ML) algorithms to identify colorimetric or textural features correlated with Hb levels, offering a rapid, cost-effective alternative to traditional blood tests [21,24]. In addition, portable, smartphone-connected hemoglobinometers allow for more precise readings using small capillary blood samples, enabling longitudinal monitoring and integration with telehealth services [25]. While these solutions offer clear advantages in terms of accessibility and ease of use, further validation and regulatory approval are necessary to ensure clinical reliability and safety.

### This Study

This pilot study explores the feasibility of using a noncontact, PPG-based mobile app, previously considered for the detection of vital signs [26,27], for Hb monitoring. The rise of noninvasive tools embodies the evolution of mobile health technologies aimed at promoting individual autonomy in health management through accessible, cost-effective, and scalable solutions, particularly suited for large-scale screening and use in resource-limited settings.

## Methods

### Study Design

This observational study evaluated the accuracy of a noncontact smartphone app, comestai.app (Come Stai S.p.A.)*,* in measuring Hb, using a standard blood reference. All app data were collected by trained personnel, and smartphone connectivity was disabled during sessions to ensure offline data acquisition. Data were securely stored using anonymized identification numbers. The study’s complete protocol has been registered on ClinicalTrials.gov (NCT06427564).

### Recruitment

Adult volunteers (aged ≥18 years, both sexes) were enrolled from outpatient clinics at Azienda Socio-Sanitaria Territoriale-Fatebenefratelli Sacco-Buzzi Hospital (Milan, Italy), Istituto di Ricovero e Cura a Carattere Scientifico MultiMedica (Milan, Italy), and Istituto di Ricovero e Cura a Carattere Scientifico Istituto Auxologico Italiano (Milan, Italy) between September 2024 and April 2025. Participants were referred for management of obesity, diabetes, or hypertension. All patients attending the clinics during the study period were consecutively approached and invited to participate. Following verbal agreement, written informed consent was obtained in accordance with ethical standards. After consent was provided, demographic and biometric data, including sex, age, weight, height, and BMI (kg/m²), were collected through direct patient interview and verified against medical records. Subsequently, clinical measurements were performed in accordance with the study protocol.

### Eligibility Criteria

To participate in the study, participants had to meet the inclusion criteria and not present any of the exclusion criteria listed in Textbox 1.

Textbox 1. **Inclusion and exclusion criteria.**

**Inclusion criteria**

- Ability to understand study information and provide written informed consent
- Participants aged between 18 and 65 years
- Capability and willingness to comply with all study-related procedures

**Exclusion criteria**

- Circulatory problems, injuries, or anatomical anomalies involving the fingers, hands, ears, forehead, skull, or any other body area essential to the study assessments
- Tattoos located in areas relevant to optical measurements that interfere with data collection
- Known hypersensitivity or allergic reactions to common medical-grade materials such as adhesives or latex used in sensor-based apps
- Any health condition that, based on the investigator’s clinical judgment, could interfere with the performance or validity of study evaluations
- General unsuitability for participation as determined by the investigator

### Measurement Procedure

Participants were seated and allowed a 2-minute rest before measurements. During testing, they faced a smartphone running comestai.app*,* which used the front-facing camera to capture facial videos. Simultaneous readings were collected for Hb over approximately 90 seconds using the app [26,27].

Ambient lighting was standardized for all remote photoplethysmography (rPPG) recordings. No medical decisions were made based on app-generated data.

A complete blood count, including Hb levels, was used as a reference for comparison with the data collected using comestai.app.

### App Description

Comestai.app uses a proprietary signal processing pipeline for rPPG via the smartphone’s front camera. The system captures facial video frames, focusing on the upper cheek, and processes red, green, and blue signals to extract subtle variations in skin coloration due to blood flow. As previously reported, the signal processing pipeline involves several sequential steps. First, raw signals are acquired using the app’s software development kit. These signals are then analyzed through rPPG techniques, which detect subtle color fluctuations in the skin caused by pulsatile blood flow and convert them into raw pulse waveforms. The resulting signal undergoes processing to reduce noise and motion artifacts, using filtering, normalization, and quality control methods. Subsequently, advanced ML, deep learning, and computer vision techniques are applied to extract relevant features from the cleaned signal. Finally, the system validates the output in real time, ensuring that only measurements with high confidence levels are reported. The app functions offline and does not transmit user data. Version 5.6.3 was used on iOS devices during this study [27].

### Statistical Analysis

Descriptive statistics for numerical variables (mean, SD, median, quartiles, and minimum-maximum) and frequencies for categorical variables were reported. Accuracy was assessed by comparing app readings with those from reference devices using mean error, mean absolute error (MAE), root mean square error, and mean absolute percentage error (MAPE).

Diagnostic performance was evaluated through sensitivity, specificity, positive predictive value, negative predictive value, accuracy, and likelihood ratios, each with 95% CIs.

Thresholds for normal physiological parameters were adopted from WHO references: Hb <12 g/dl for women and Hb <13 g/dl for men [1]. In our study, the reference to WHO diagnostic thresholds was used solely as a benchmark to contextualize the results within widely recognized clinical standards, not to suggest diagnostic use, and the app should be regarded as a proof-of-concept wellness app.

Correlations between app and reference data were calculated using Pearson *r*, with results visualized via regression plots including *R*² values. Agreement was further assessed with Bland-Altman analysis. All analyses were performed using Microsoft Excel (with Solver and Data Analysis ToolPak) and partially verified in R software (version 4.3.3; R Foundation for Statistical Computing) for cross-validation.

### Ethical Considerations

To enhance the methodological quality, the protocol has been developed in accordance with the SPIRIT (Standard Protocol Items: Recommendations for Interventional Trials) guidelines for clinical trial protocols (Checklist 1).

## Results

A total of 555 participants (female: n=313, 56.4%; male: n=242, 43.6%) were enrolled. The sample was well balanced in terms of sex. Participants were distributed across 3 age groups: 8.3% (n=46) were aged between 18 and 35 years, 26.9% (n=149) were aged between 36 and 55 years, and 64.8% (n=360) were older than 55 years. The mean BMI was 30.69 (SD 7.34) kg/m², indicating that the study population predominantly comprised participants with overweight or obesity, as expected for the enrolled population. On the basis of blood Hb values, a prevalence of 10.27% (n=57) anemia was observed.

Table 1 summarizes the descriptive data and performance metrics of Hb values recorded using the mobile app compared to those obtained via standard laboratory methods. The app achieved an MAE of 1.46, MAPE of 11.26, a mean error of −0.67, and root mean square error of 1.88.

**Table 1.** Descriptive statistics and agreement metrics for hemoglobin values recorded via the mobile app and laboratory reference device.

Parameters	Mean (SD)	Median (IQR)	Mean absolute error	Root mean square error	Mean error	Mean absolute percentage error	Pearson correlation coefficient
App	13.37 (1.10)	13.30 (12.70-14.10)	1.46	1.88	−0.67	11.26	0.12
Laboratory	14.04 (1.51)	14.00 (13.10-15.00)	1.46	1.88	−0.67	11.26	0.12

**Figure 1.**
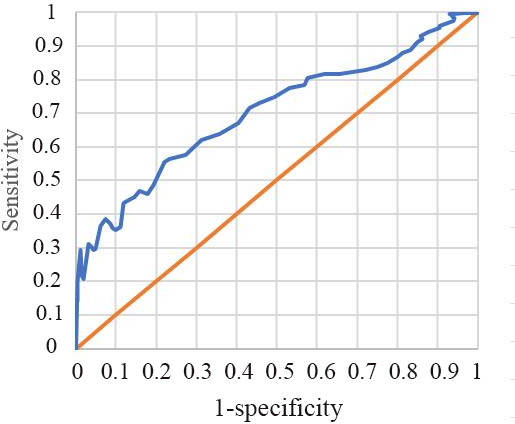
Receiver operating characteristic curve. The area under the curve was 0.701 (95% CI 0.655-0.745).

In Table 2, the diagnostic accuracy of Hb detection using the app was assessed against conventional measurements. Sensitivity, specificity, positive predictive value, negative predictive value, positive likelihood ratio, negative likelihood ratio, and accuracy were calculated. The method showed good specificity (88.9%), but low sensitivity (6.4%), with an accuracy of 75%. As represented in Figure 1, the area under the curve was 0.701 (95% CI 0.655-0.745).

**Table 2.** Accuracy of vital parameters using the mobile app and the conventional method.

	Hemoglobin^a^
Specificity	0.889
Sensitivity	0.064
Positive predictive value	0.105
Negative predictive value	0.823
Accuracy	0.750
Positive likelihood ratio	0.577
Negative likelihood ratio	1.053

a Normal hemoglobin values are ≥12 g/dl for women and ≥13 g/dl for men.

**Figure 2.**
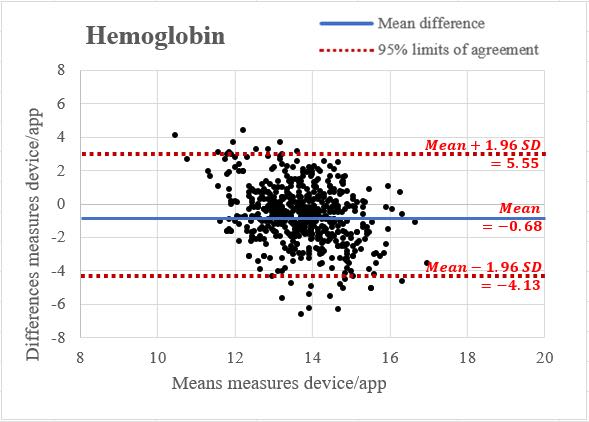
Bland-Altman plot for hemoglobin measurements comparing the mobile app with the standard method. The plot includes a central horizontal line indicating the average difference between the 2 measurement methods. Two additional horizontal lines are drawn to show the limits of agreement, defined as the mean difference (SD 1.96) times the SD of the differences. This range encompasses 95% of the differences between the methods, providing a visual representation of their agreement.

The Bland-Altman plot in Figure 2 evaluated the agreement between the app-based and laboratory-based Hb measurements. Most data points fell within the 95% CI, with the mean difference between the 2 methods being −0.70 g/dL. The clustering of points around the mean and a narrowing spread at higher Hb levels suggest a generally good agreement despite some systematic underestimation by the app.

## Discussion

### Principal Findings

Comestai.app offers an innovative approach to wellness monitoring by providing noninvasive Hb estimation using the smartphone’s front-facing camera. This study assessed the accuracy and diagnostic reliability of the app compared to standard laboratory methods. While the app slightly underestimated Hb values on average, overall agreement was observed, with most data falling within acceptable limits for nonmedical apps. These findings suggest that the app may serve as a useful tool for preliminary screening and self-monitoring, especially in wellness contexts where invasive methods are impractical or unavailable.

As indicated by the WHO [1], anemia is one of the most widespread health conditions globally, negatively affecting energy levels, productivity, and quality of life. Therefore, Hb monitoring is a fundamental tool in health promotion, especially in settings where access to conventional diagnostic tools is limited [28].

Conventional Hb measurement using venous blood is considered highly accurate and serves as the clinical gold standard. However, it involves discomfort due to needle pricks, requires trained personnel and expensive equipment, and causes delays in result reporting [5,7,8]. In contrast, noninvasive or minimally invasive POC devices offer quicker results, greater patient comfort, and ease of use, particularly in resource-limited settings. These devices enhance portability and patient satisfaction but may be less accurate and require regular calibration, while also being sensitive to environmental conditions such as light and temperature [10,11].

In this context, mobile health technologies have emerged as strategic tools for wellness-oriented self-assessment [29-31], including the monitoring of Hb levels [21,32]. Although not classified as medical devices, these solutions can facilitate individual health tracking and support the early identification of deviations from physiological norms, thereby contributing to improved self-management and preventive health care practices.

Mobile devices and digital apps, such as comestai.app, now allow users to track their blood levels directly from home, without the need for additional equipment or clinical infrastructure, thereby reducing the need for frequent medical visits [33-35]. This approach improves accessibility in remote or underserved areas, reduces costs compared to traditional laboratory testing, and fosters patient autonomy in managing anemia or chronic health conditions more proactively. It is particularly relevant for older individuals [36,37] who are more susceptible to anemia and often face mobility challenges, thus highlighting the importance of regular, noninvasive Hb monitoring.

Compared to other noncontact or smartphone-based technologies, comestai.app shows performance that is within the expected range. For example, HemaApp, a smartphone-based Hb monitoring tool proposed by Wang et al [38], achieved an accuracy of ±1.26 g/dL with a correlation coefficient of 0.82 when compared to standard blood tests. HemaApp required additional light sources for optimal performance, whereas comestai.app works using only ambient light and a smartphone camera, thus improving ease of use and accessibility, though possibly at the cost of slightly lower sensitivity.

A study published by Mannino et al [21] demonstrated a fingernail image–based system with 95% limits of agreement of ±2.4 g/dL and minimal bias, showing high sensitivity (up to 97%) in detecting anemia at a standard clinical threshold. In comparison, comestai.app showed excellent specificity but lower sensitivity, resulting in an overall good level of accuracy. This discrepancy is likely due to differences in the study population and the relatively small proportion of participants with low Hb levels in our sample. Such imbalance may have limited the model’s exposure to pathological cases, reducing its diagnostic power for anemia detection.

Other promising rPPG-based models, such as one validated in a clinical setting in Singapore, reported a MAPE of 8.52% and a mean difference of 0.23 g/dL between predicted and laboratory Hb values. In our case, the MAPE was slightly higher, possibly reflecting differences in signal processing techniques, but still within the range reported for wellness-oriented apps [39,40]. The Bland-Altman analysis from our study revealed a narrow spread and clustering around the mean at higher Hb levels, suggesting greater measurement stability in individuals with normal or elevated values.

In our study, the Pearson correlation coefficient between Hb values estimated by the app and those obtained through standard clinical methods was low. However, a strong linear correlation is not necessarily required for the intended purpose of the app, which is not to reproduce exact laboratory values but rather to identify individuals at potential risk of anemia using threshold-based approaches. Even with limited absolute agreement, the app may still discriminate between individuals above or below clinically relevant cutoffs (eg, the WHO anemia threshold), highlighting potential utility despite weak linear correlation. Moreover, as previously reported for other vital parameters [41], improvements in the *P* value can be achieved by refining signal processing techniques and incorporating multiple facial regions of interest. These approaches could similarly contribute to improving the accuracy of Hb measurements. In addition, we acknowledge the presence of a systematic bias of −0.70 g/dL, which may be clinically significant and needs to be addressed in terms of both its implications and the possibility of algorithmic calibration in future versions. Therefore, future developments will prioritize optimizing the signal processing pipeline and enhancing robustness against confounding factors such as lighting variability and participant motion, with the aim of improving the accuracy and reliability of noninvasive Hb estimation.

One key advantage of comestai.app is its entirely contactless approach, making it suitable for frequent home use without additional equipment. This is especially valuable in wellness contexts and for populations with limited access to health care. In particular, it may offer a valuable alternative for specific populations, such as older individuals, who may require more frequent monitoring due to their increased susceptibility to anemia and reduced physiological reserves [42-44], as well as other at-risk groups including pregnant women [45-48], patients with chronic diseases (such as chronic kidney or inflammatory conditions) [49-54], and individuals with malabsorptive gastrointestinal disorders [55-57]. Looking ahead, such user-friendly, noninvasive technologies could contribute meaningfully to broader public health strategies by enabling early identification of at-risk individuals, promoting health awareness, and reducing reliance on clinical resources for routine screening. Future algorithm updates could substantially improve sensitivity and correlation, as previously demonstrated in research on digital monitoring of vital parameters [41]. With further improvements and appropriate adaptations, the tool may also become suitable for use in children, who often face logistical and emotional challenges with traditional blood sampling [58-60].

It is important to note that, unlike clinical-grade tools, comestai.app is not intended for diagnostic use. Its classification as a wellness device aligns with international standards that tolerate higher error margins for nonmedical apps, and the reported low sensitivity should be interpreted in light of the preliminary nature of this pilot study, the small sample size, and the limited number of patients with anemia. Nevertheless, given that anemia often presents without symptoms until reaching moderate to severe stages, tools capable of early detection, even with limited precision, can still offer significant public health value by promoting timely medical consultation.

In particular, as described by Saleh et al [61], the performance of these tools can be significantly enhanced by developing ML algorithms trained on clinical datasets and PPG signals. This strategy enables the differentiation between normal and anemic conditions via multistep processing pipelines involving data labeling, signal preprocessing, feature extraction, and model training. The referenced study showed that by combining red and infrared PPG data with a rich set of extracted features, anemia could be classified with up to 100% accuracy using a 5-fold cross-validation approach. Additionally, Mannino et al [32] showed that personalization of the app’s artificial intelligence–augmented algorithm enhances self-monitoring of Hb levels, leading to an improvement in the app’s MAE. These results highlight the potential of ML-augmented, PPG-based systems to serve as scalable, effective, and fully noninvasive solutions for anemia screening.

This study presents several limitations that should be acknowledged when interpreting the findings. First, although the sample included participants with different Hb levels, the prevalence of clinically significant anemia was relatively low (57/555, 10.27%), which likely reduced the statistical power and contributed to the limited sensitivity in detecting low Hb values. No formal power calculation was performed, further constraining the robustness of the diagnostic accuracy assessment. Moreover, recruitment from specialized outpatient clinics focusing on obesity, diabetes, and hypertension introduces selection bias, limiting the generalizability of the results to broader populations. Future studies should include a more balanced sample, particularly with a higher representation of individuals living with anemia. Second, the measurements were conducted in controlled settings, which may not fully capture the variability and challenges of real-world conditions. Factors such as ambient lighting, facial positioning, skin tone variation, and user movement can introduce noise and affect the reliability of Hb estimation in everyday use. Finally, no subgroup analyses were performed to evaluate performance across different health conditions; such stratified analyses would be necessary to assess the generalizability of the findings and to identify potential performance disparities in clinically relevant subpopulations.

Further research, including longitudinal studies, field-based validation trials, and large-scale deployment of ML-augmented PPG systems, is needed to enhance algorithm robustness, improve clinical utility, and confirm effectiveness across diverse populations and real-world settings. Such efforts will be essential to ensure generalizability, address potential sources of bias, and support the integration of these technologies into routine screening and preventive care frameworks.

### Conclusions

This study explores the feasibility of a noncontact PPG-based mobile app for noninvasive Hb monitoring, demonstrating how comestai.app represents an innovative approach to wellness monitoring through Hb estimation using the smartphone’s front-facing camera. While not yet suitable for clinical diagnosis due to limited sensitivity, its high specificity and ease of use support its role as a valuable tool for self-monitoring. It could also be useful in large-scale initial screening programs to identify individuals who may benefit from further clinical evaluation. Continued development, including algorithmic refinement and larger-scale validation in diverse populations, will be key to enhancing accuracy and broadening its utility. By leveraging the ubiquity of smartphones, comestai.app contributes meaningfully to the democratization of health monitoring and the promotion of proactive self-care.

## Supplementary material

10.2196/78820 Checklist 1 SPIRIT checklist.

## Abbreviations

Hb: hemoglobin
MAE: mean absolute error
MAPE: mean absolute percentage error
ML: machine learning
POC: point-of-care
PPG: photoplethysmography
rPPG: remote photoplethysmography
SPIRIT: Standard Protocol Items: Recommendations for Interventional Trials
WHO: World Health Organization
